# FOXM1 Modulates Cisplatin Sensitivity by Regulating EXO1 in Ovarian Cancer

**DOI:** 10.1371/journal.pone.0096989

**Published:** 2014-05-13

**Authors:** Jinhua Zhou, Yunfei Wang, You Wang, Xia Yin, Yifeng He, Lilan Chen, Wenwen Wang, Ting Liu, Wen Di

**Affiliations:** 1 Department of Obstetrics and Gynecology, Renji Hospital, School of Medicine, Shanghai Jiao Tong University, Shanghai, China; 2 Shanghai Key Laboratory of Gynecologic Oncology, Shanghai, China; 3 Focus Construction Subject of Shanghai Education Department, Shanghai, China; 4 Shanghai Health Bureau Key Disciplines and Specialties Foundation, Shanghai, China; 5 Department of Obstetrics and Gynecology, The First Affiliated Hospital of Soochow University, Suzhou, Jiangsu, China; University of Texas Health Science Center at San Antonio, United States of America

## Abstract

Cisplatin is commonly used in ovarian cancer chemotherapy, however, chemoresistance to cisplatin remains a great clinical challenge. Oncogenic transcriptional factor FOXM1 has been reported to be overexpressed in ovarian cancer. In this study, we aimed to investigate the potential role of FOXM1 in ovarian cancers with chemoresistance to cisplatin. Our results indicate that FOXM1 is upregulated in chemoresistant ovarian cancer samples, and defends ovarian cancer cells against cytotoxicity of cisplatin. FOXM1 facilitates DNA repair through regulating direct transcriptional target EXO1 to protect ovarian cancer cells from cisplatin-mediated apoptosis. Attenuating FOXM1 and EXO1 expression by small interfering RNA, augments the chemotherapy efficacy against ovarian cancer. Our findings indicate that targeting FOXM1 and its target gene EXO1 could improve cisplatin effect in ovarian cancer, confirming their role in modulating cisplatin sensitivity.

## Introduction

Ovarian cancer is the most lethal gynecologic malignancy in the world, with 225,500 new cases and 140,200 deaths estimated for 2008[1]. Most women with epithelial ovarian cancer (EOC) present with advanced disease (stage III or IV) at the time of diagnosis. Current standard treatment of ovarian cancer, in both early and advanced stages, consists of complete cytoreductive surgery followed by chemotherapy, usually based on a platinum and taxane doublet [2]. But the development of chemoresistance still presents a major impediment for the successful treatment. Most patients succumb to chemoresistance and relapse, and the overall 5-year survival rate is about 31%[3]. A better understanding of the molecular basis of cisplatin resistance may lead to new antitumor strategies that will sensitize unresponsive ovarian cancers to cisplatin-based chemotherapy.

Mammalian transcription factor Forkhead Box M1 (FOXM1) belongs to a large family of Forkhead transcription factors. Forkhead family members are involved in a wide range of biological processes including embryogenesis, proliferation, differentiation, apoptosis, transformation, tumorigenesis, longevity, and metabolic homeostasis[4]. Unlike the other FOX-transcription factors, FOXM1 is associated with cell proliferation and is overexpressed in cancer. For example, gene expression profiles in carcinomas, including prostate, breast, lung, ovary, colon, pancreas, stomach, bladder, ovarian, liver, and kidney, revealed that FOXM1 is overexpressed in all carcinomas [5]–[9]. Overexpression of FOXM1 in various tumors indicates a strong dependence of the tumor cells on FOXM1[10]. Moreover, in ovarian cancer, the integrated pathway analysis showed that FOXM1 transcription factor network is significantly altered in 87% of high-grade serous ovarian cancer[11]. FOXM1 promotes cell proliferation, migration and invasion in ovarian cancer[12]. FOXM1 has also been demonstrated to play a crucial role in drug responsiveness and resistance. For instance, it has been shown that deregulated FOXM1 expression can confer resistance to chemotherapeutic drugs, such as cisplatin and epirubicin[13], and protect cancer cells against DNA-damage induced cell death in breast cancer[14]. However, it remains elusive whether the FOXM1 play a similar role responsible for conferring cisplatin resistance in ovarian cancer.

EXO1 is a protein with 5′ to 3′ exonuclease activity as well as an RNase H activity, which interacts with Msh2 and which is involved in mismatch repair and recombination[15], [16]. Recent study shows that EXO1 contributes to the induction of DNA damage checkpoints and participates in DNA damage repair [17], [18].

In the present study, we provide the evidences that FOXM1 and its direct downstream DNA repair gene EXO1 might play in increasing the survival of ovarian cancer cells after cisplatin treatment, and targeting FOXM1/EXO1 axis can sensitize ovarian cancer cell to cisplatin treatment.

## Materials and Methods

### Ethics Statement

The protocols for handling paraffin-embedded ovarian cancer specimens and analyzing patient data were approved by the ethical committees of Renji Hospital, Shanghai Jiao Tong University, China. Written informed consents were signed by each enrolled patient if she was still alive or by her first-degree relative if she has died. All tissue samples were registered by a case number in the database with no patient names or personal information indicated.

### Immunohistochemistry

The paraffin-embedded tissue samples were collected from 20 women with primary epithelial ovarian cancer, stagesIIto IV, who had undergone initial surgery at the department of obstetrics and gynecology, Renji Hospital, School of Medicine, Shanghai Jiao Tong University between 2005–2008. The slides were deparaffinized, rehydrated and placed into citric acid buffer (pH 6.0, 0.1 M) for heating for 10 min. The endogenous peroxidase activity was then blocked by incubation with 3% H_2_O_2_ for 10 min. Afterwards, sections were incubated with blocking buffer (Beyotime, China) for 1 h and then incubated overnight at 4°C with FOXM1 antibody (1∶50, Santa Cruz). Following a 10-min incubation of biotinylated second antibody, the slides were again incubated with streptavidin-peroxidase under the same condition. The immunoreaction was then visualized by incubation with diaminobenzidine chromogen (DAB, Maixin-Bio, China) for 5 min. Finally, the slides were counterstained with hematoxylin, dehydrated, cleared and mounted. Negative controls were incubated in blocking buffer alone. These results were only considered if these control samples demonstrated a negative staining.

### Cell lines and Culture

The human ovarian cancer cell lines A2780 and SKOV3 were purchased from the Cell Bank of the Chinese Academy of Science (Shanghai, China). Both cell lines were cultured in RPMI 1640 (Hyclone, USA) supplemented with 10% (v/v) fetal bovine serum, 100 units/ml penicillin and 100 g/ml streptomycin and cells were maintained at 37°C in a humidified atmosphere containing 5% CO2/95% air.

### siRNA and plasmid transfection and co-transfection

siRNA duplexes were prepared by RiboBio(Guangzhou,China). SiRNA The sequence of siRNAs were as follows: FOXM1 siRNA-1: 5'- GCCAAUCGUUCUCUGACAGAATT-3', siRNA-2: 5'- GGACCACUUUCCCUACUUUUUTT-3'[19]. EXO1 siRNA-1: 5'-CAAGCCUAUUCUCGUAUUUTT-3', siRNA-2: 5'-UAGUGUUUCAGGAUCAACAUCAUCU-3'[18]. The sequence of negative control (NC) was: 5′-UUCUCCGAACGUGUCACGUTT-3'. Transfection or co-transfection of siRNA and plasmid was performed according to the manufacturer's protocol of lipofectamin (Invitrogen).

### Real-time PCR

Total RNA was extracted using Trizol (Invitrogen), and cDNA was synthesized using PrimerScript RT reagent Kit (Takara). For real-time quantative PCR, equal amount of cDNA were added to SYBR premix EX Taq II (Takara) and run in Stepone real-time PCR system (Applied Biosystem). The cycling program was 95°C for 5 s and 60°C for 30 s. Each sample was assayed in triplicates, and β-actin was used as an endogenous control. The forward and reverse primers used were as follows: FOXM1: 5′- GGAGCAGCGACAGGTTAAGG-3′ and 5′- GTTGATGGCGAATTGTATCATGG-3′, EXO1: 5′- CCTCGTGGCTCCCTATGAAG-3′ and 5′- AGGAGATCCGAGTCCTCTGTAA-3′. PLK4: 5′- AAGCTCGACACTTCATGCACC-3′ and 5′- GCATTTTCAGTTGAGTTGCCAG-3′. XRCC1: 5′- CCTTTGGCTTGAGTTTTGTACG-3′ and 5′- CCTCCTTCACACGGAACTGG-3′. BRCA2: 5′- TGCCTGAAAACCAGATGACTATC-3′ and 5′- AGGCCAGCAAACTTCCGTTTA-3′. Rad51: 5′- CAACCCATTTCACGGTTAGAGC-3′ and 5′- TTCTTTGGCGCATAGGCAACA-3′. β-actin: 5′- CATGTACGTTGCTATCCAGGC-3′ and 5′- CTCCTTAATGTCACGCACGAT-3′.

### Clonogenic assay

After transfection of NC or gene-specific siRNA, cells were subjected to the indicated concentration of cisplatin for 1 h. Then cells were resuspended in fresh complete medium and plated in 6-well cell culture plate at the density of 500 cells/well. Following incubation at 37°C in a humidified atmosphere containing 5% CO2/95% air for 8–10 days, media was changed every 3 days. At the end of culture, cells were stained with 1% methylene blue in 50% methanol for 20 min, washed with water, and colonies (≥50 cells) were counted.

### Western blot

Cells were lysed in RIPA buffer with supplement of PMSF protease inhibitor, followed by centrifugation at 14000 g for 10 min. At the end of centrifugation, cell lysates were collected and protein concentration of cell lysates was measured. Equal amount of proteins (10–20 µg) were resolved by SDS-PAGE, and transferred to PVDF membrane (Millipore). The blots were then incubated with primary antibodies in 5% bovine serum albumin/Tris-buffered saline Tween-20 at 4°C overnight, followed by incubation with secondary antibodies at room temperature for 1 h. The protein signals were detected by Odessey scanner. The antibodies used in this study included: human FOXM1 (1∶100, Santa Cruz Biotechnology), phospho-histone H2A.X (γH2AX, 1∶1000, Cell Signaling Technology), caspase-3 (1∶1000, Cell Signaling Technology), EXO1 (1∶100, Thermo Fisher Scienfitic), β-actin (1∶2000, Abcam).

### Cell viability assay

Cell viability was measured by Cell Counting Kit-8 assay (Dojindo Molecular Technologies). Briefly, cells were plated at a density of 5×10^3^ cells/well on 96-well plates and subjected to different treatment. Following 48 h incubation at 37°C in a humidified atmosphere containing 5% CO_2_/95% air, the samples were incubated for another 2 h with CCK8 reagent. The absorbance was determined at 450 nm using FLx800 Fluorescence Microplate Reader (Biotek).

### γH2AX immunofluorescent staining

A2780 and SKOV3 cells were transfected with NC or gene-specific siRNA. After 48 h, cells were then treated with the indicated concentration of cisplatin for 1 h, and fresh media were changed. 24 hours later, cells were subjected to anti-γH2AX (Ser139) staining. Briefly, cells were fixed with 10% formalin for 15 min, then permeabilized with 0.1% Triton-100 in 10% FCS for 10 min. Samples were blocked with 5% goat serum in 10% FCS for 1 h and then incubated overnight with the primary rabbit anti-γH2AX (Ser139;1∶400; Cell Signaling). Following washes with PBS, secondary goat anti-rabbit IgG-TRITC (1∶400; Sigma-Aldrich) was added to the samples for 1 h. Cells were counterstained with DAPI before mounting. Images were captured using a Laser Scanning Confocal microscope TCS SP5 (Leica). For foci quantification, cells with greater than 10 foci were counted as positive according to the standard procedure[20]. Experiments were repeated in triplicate.

### Flow cytometry

Apoptosis was exmamined by flow cytometric analysis of Annexin V and PI staining (BD) according to the manufacturer's protocol. Briefly, after the indicated treatment, cells were resuspended at a concentration of 1×10^6^ cells/ml, then 5 µl of FITC annexin V and 5 µl of PI were added to 1×10^5^ cells (100 µl) and the cells were incubated at room temperature for 15 min. After incubation, cells were analyzed by flow cytometer FC500/FC500-MPL (Beckman Coulter). For cell cycle analysis, cells were trypsinized, pelleted, and then resuspended in propidium iodide solution (50 µg/ml propidium iodide, 0.1 mg/ml RNaseA, and 0.05% Triton-X). All reagents were purchased from Sigma. After 40 min of incubation, cells were analyzed by FC500/FC500-MPL.

### Chromatin immunoprecipitation assay

Chromatin immunoprecipitation (ChIP) experiments were performed as previously described [21]. 24 hours after cisplatin treatment, cells were fixed in 1% formaldehyde for 10 min to allow crosslinking and then quenched with glycine. Cells were collected and lysed in SDS lysis buffer. Lysate was sonicated, pre-cleared, incubated with antibodies, and collected with Protein-A+G agarose/Salmon Sperm DNA. DNA-protein cross-links were reversed and chromatin DNA was purified and subjected to PCR analysis. The primers 5'–AAA TCT GGC AAC CCT ACC TCA-3' and 5'-TTA AGT GTG CCT GTC AGT TCC-3' were used to amplify the EXO1 FHRE1-containing region (−1934/−1575), and the primers 5'-CAA TTT CGA TTT GTA GAG GCA AC-3' and 5'-CGG CTT CCA ACT CAT AGG GT-3' were used to amplify the FHRE2-containing region (−459/−74). After amplification, PCR products were resolved on agarose gel visualized by GelRed (Biotium).

### Promoter reporters and luciferase assays

The EXO1 promoter region was PCR-amplified from genomic DNA extracted from A2780 cells using forward and reverse primers containing NheI and HindIII restriction sites (5'-CTA GCT AGC AGG ACC AAA GAG CCA TCA CA-3' and 5'-CCC AAG CTT CAC GGG TAA CTT GCC TAC ACA 3'). After restriction digestion, the fragment was cloned in the pGL-3 basic reporter gene vector to generated the EXO1 promoter construct, pGL3-FHER2 promoter construct −490/−148 was cloned by PCR (primers: 5'- CTA GCT AGC AAA GAA CCC AGC GTG AAC TGA-3', 5'- CCC AAG CTT CAC GGG TAA CTT GCC TAC ACA 3'). Putative Forkhead site mutagenesis was performed using a site-directed mutagenesis kit (ExCell Biology). pGL3-Basic, pGL3-EXO1, wild-type pGL3-FHRE2, wild-type pGL3-FHRE2 plus FOXM1 siRNA, and mutant pGL3-FHRE2 were transfected to cells respectively. Transfected cells were treated with cisplatin for 24 h and their luciferase activities were measured by luciferase assay system (Promega).

### Statistical analysis

We used SPSS19.0 software to calculate standard deviations and statistically significant differences between samples. The asterisks in each graph indicate statistically significant changes, with *P* values calculated by the Student *t* test as follows: *, *P*<0.05; **, *P*≤0.01; ***, *P*≤0.001. *P* values of <0.05 were considered statistically significant.

## Results

### 1. FOXM1 expression was up-regulated in cisplatin resistant ovarian cancer tissues and cells

**Figure 1:**
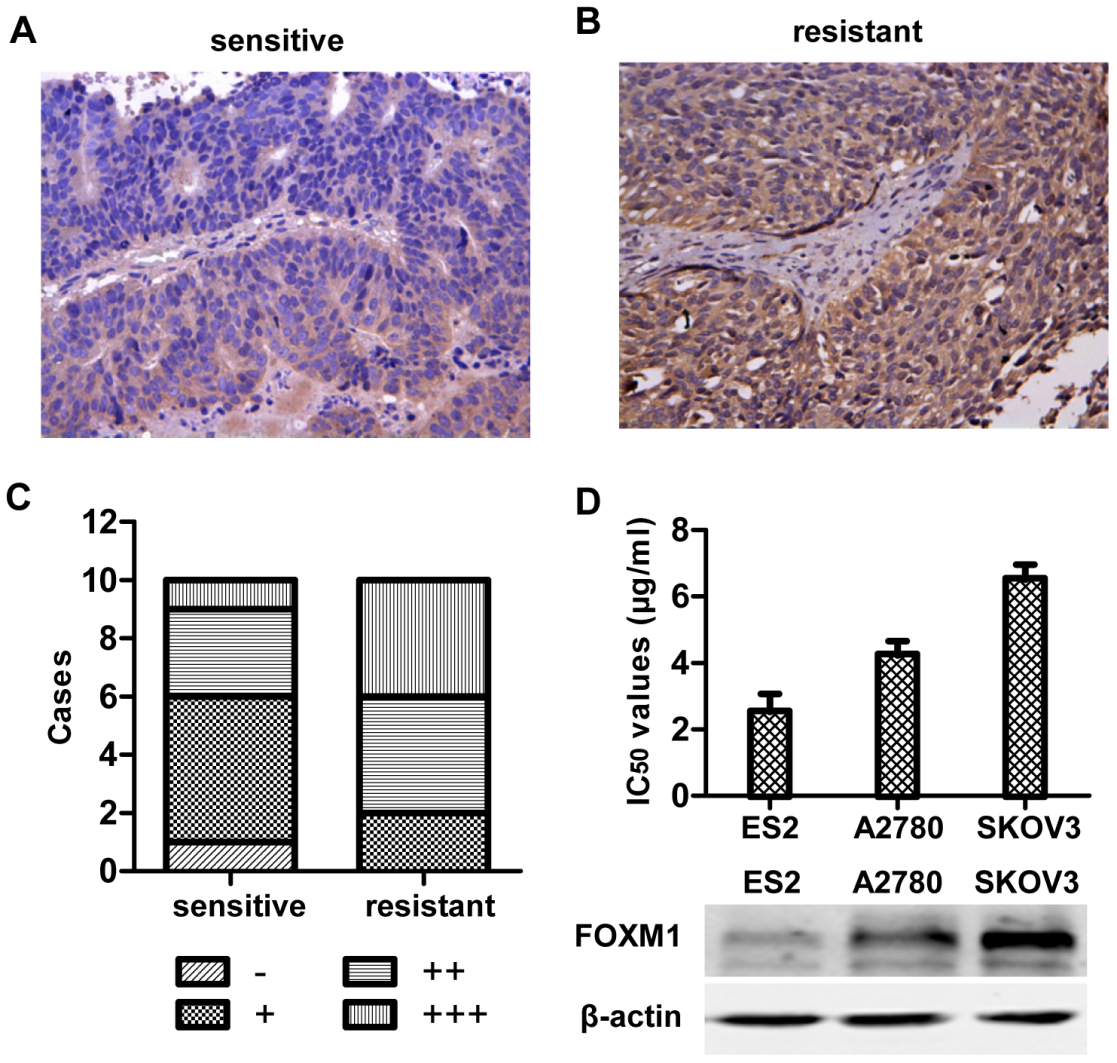
FOXM1 is upregulated in chemoresistant ovarian cancer tissue. (A) Representative FOXM1 immunostained section is shown from the chemosensitive patient group, (B) Representative FOXM1 immunostained section is shown from the chemoresistant patient group. (C) The numbers of cases with the indicated level of FOXM1 expression in chemosensitive and chemoresistant group are shown.(D) The IC_50_ values of different cell lines were shown in the upper panel, and the expression of FOXM1 in cell lines were shown in the lower panel.

Previously, it has been shown that FOXM1 is overexpressed in ovarian cancer, and that FOXM1 overexpression was significantly correlated with high-grade ovarian cancers, indicating that FOXM1 may play an oncogenic role in ovarian cancer [12]. Thus far, the role of FOXM1 in cisplatin resistance of ovarian cancer has not been elucidated. To investigate the expression of FOXM1 in cisplatin sensitive or resistant ovarian cancer, ovarian cancer tissue samples were obtained from 10 women with recurrent epithelial ovarian cancer in 6 months after standard therapy, and other 10 women who were chemosensitive. All patients received optimal cytoreductive surgery followed by 6 cycles of systemic chemotherapy with the combination of cisplatin and paclitaxel. Among the 10 chemosensitive patients, 7 of them recurred after 12 months and 3 of them did not recur so far. All the slides were from the ovarian cancer tissues resected in the initial operation. The paraffin-embedded slides were immunohistochemically stained with FOXM1 antibody and representative stained slides were shown in Fig.1A and Fig.1B. Moderate to high FOXM1 expression were detected in as much as 8 of 10 resistant cases, but only in 4 of 10 sensitive cases (Fig.1C). Next, we compared cisplatin resistance and FOXM1 expression in three cell lines. SKOV3, which showed highest expression of FOXM1, was also the most resistant to cisplatin, while ES2, which was the most sensitive to cisplatin, expressed FOXM1 at the lowest level (Fig.1D). These results indicate that cisplatin resistant ovarian cancer exhibits higher level of FOXM1 expression compared to cisplatin sensitive ovarian cancer.

### 2. FOXM1 is up-regulated in ovarian cancer cells after cisplatin treatment

**Figure 2:**
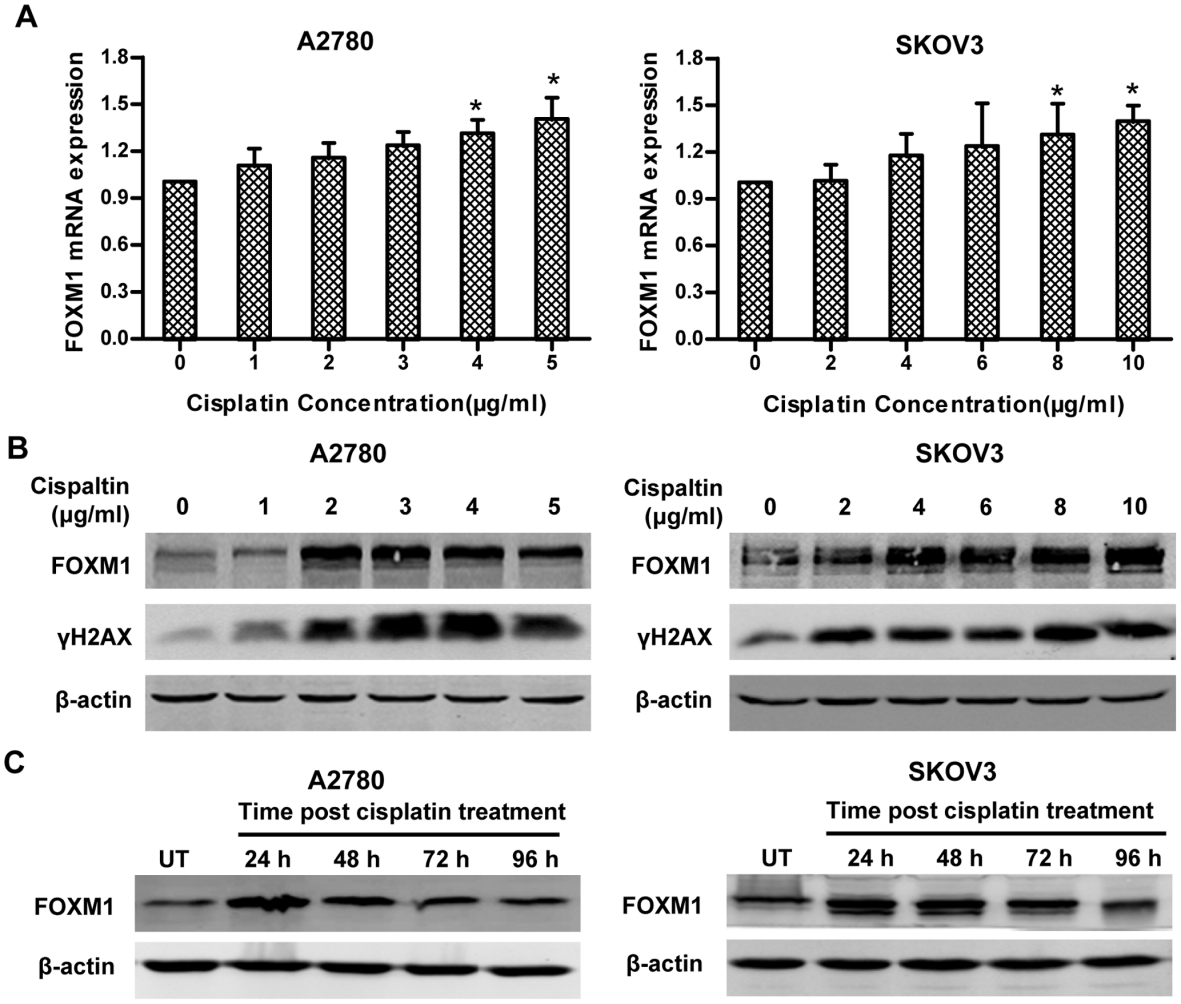
Cisplatin induces FOXM1 expression in ovarian cancer cells. (A) A2780 and SKOV3 cells were treated with the indicated concentration of cisplatin for 24 h. After treatment, FOXM1 mRNA transcription levels were determined by real-time PCR and β-actin was used as an endogenous control. Each column and bar represents mean±s.d. of triplicate determinations. (B) Cell lysates were prepared after the same treatment as in (A), resolved by SDS-PAGE and subjected to immunoblotting analysis using anti-FOXM1, anti-γH2AX, or anti-β-actin, respectively. (C) A2780 and SKOV3 cells were treated with 2 µg/ml and 4 µg/ml cisplatin for 12 h respectively, then were cultured in fresh complete medium for indicated time points (the time when complete medium was added was set as 0 h). Western blot was performed to determine the expression of FOXM1 and β-actin.

To further determine the expression pattern of FOXM1 in ovarian cancer cells, we treated A2780 and SKOV3 with different concentrations of cisplatin, and discovered the mRNA level of FOXM1 increased only slightly after cisplatin treatment (Fig.2A). We found, however, that FOXM1 protein remarkably increased in a concentration and time dependent manner in both cell lines, and corresponded to the level of γH2AX (Fig.2B and Fig.S1), which is the gold standard of DNA damage quantification[22]. We also performed ON/OFF treatment of cisplatin, elevated level of FOXM1 protein was observed in both cell lines at 24 hours post treatment, and the effect sustained till 96 hours (Fig.2C). Since the increased level of FOXM1 protein does not correspond to the increased level of FOXM1 mRNA, it was possible that elevated FOXM1 protein was largely mediated by protein stabilization [23].

### 3. Targeting FOXM1 increases cisplatin sensitivity in ovarian cancer

**Figure 3:**
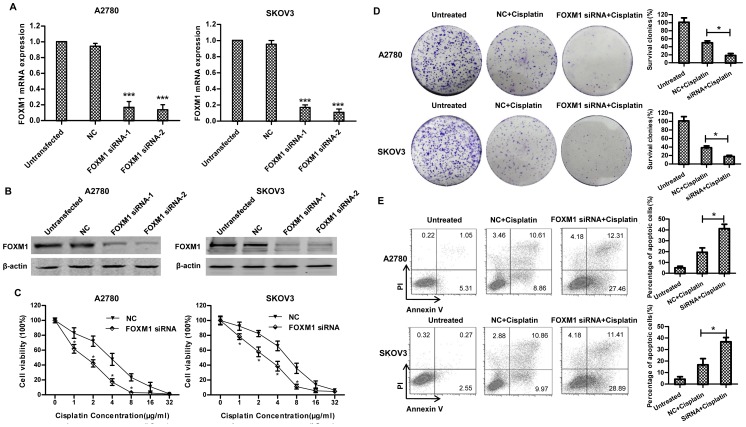
Targeting FOXM1 increases cisplatin sensitivity in ovarian cancer. A2780 and SKOV3 cells were transiently transfected with siRNA (100 nM) directed against FOXM1. Control cells were left untransfected, or negative control siRNA(100 nM)-transfected. 48 h after transfection, FOXM1 mRNA level (A) and protein level (B) was determined by real-time PCR and western blotting, respectively. Each column and bar represents mean±s.d. of triplicate determinations. (C) 48 h after transfection, A2780 and SKOV3 cells were treated with increasing concentrations of cisplatin for another 48 h and their rates of viability were measured by CCK8 and compared to cells without cisplatin treatment. (D) 48 h after siRNA transfection, A2780 and SKVO3 cells were treated for 1 h with 1 µg/ml and 2 µg/ml cisplatin, respectively. Then cells were plated in 6-well plate, colonies were stained and counted after incubation for 8–10 d. Results shown are representative of three independent experiments. The graphs provide quantification as a percentage of the non-treated wells. Each column and bar represents mean±s.d. of triplicate determinations. (E) 48 h after transfection, A2780 and SKOV3 cells were treated for another 48 h with 1 µg/ml and 2 µg/ml cisplatin, respectively. After treatment, apoptosis was determined by flow cytometric analysis of Annexin V and PI staining. The right panel shows means±s.d. of three independent experiments.

**Figure 4:**
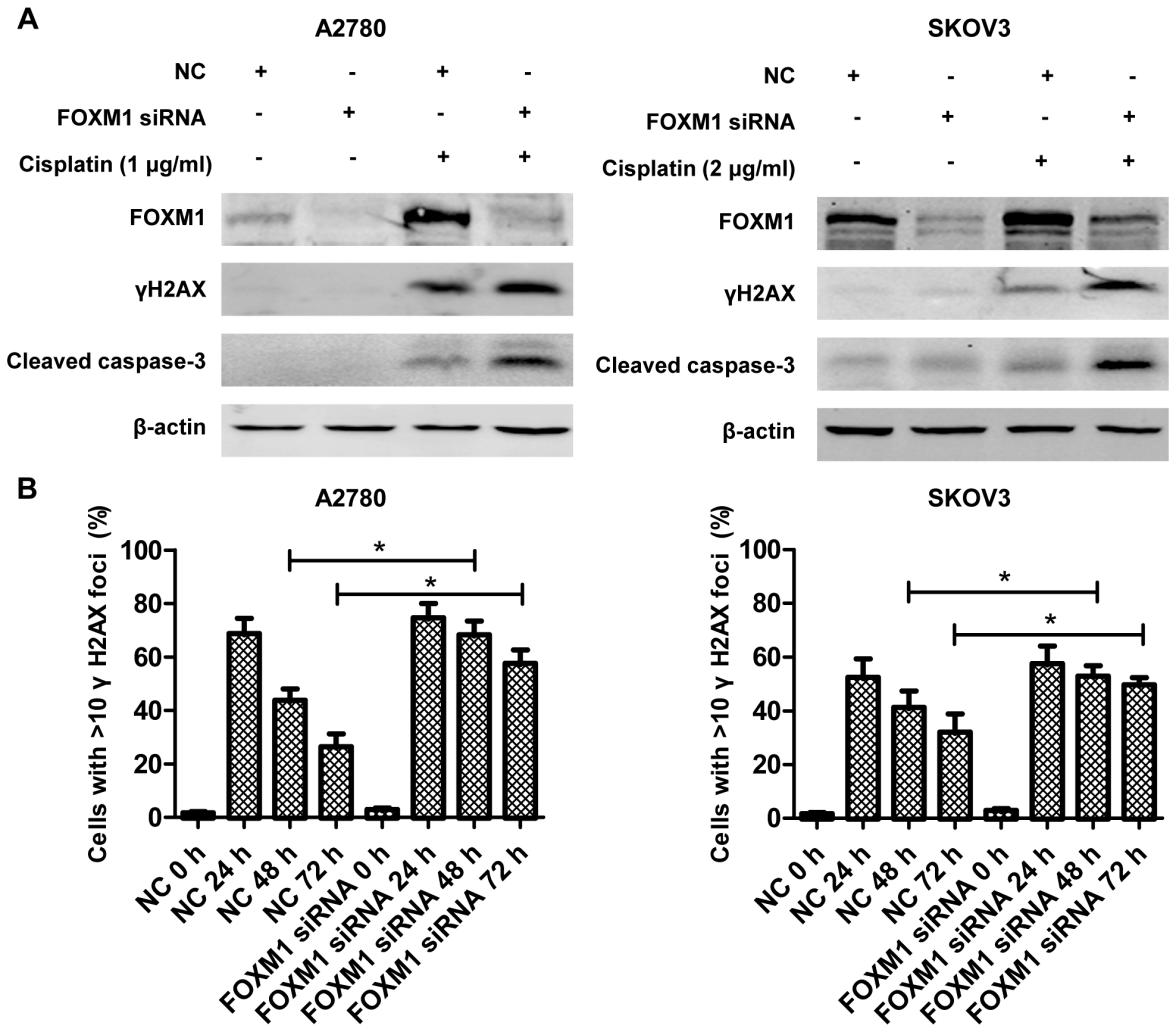
FOXM1 knocking-down leads to DNA repair deficiency. (A) 48 h after transfection, A2780 and SKOV3 cells were treated for 24 h with 2 µg/ml and 4 µg/ml cisplatin, respectively. After treatment, cell lysates were prepared, resolved by SDS-PAGE and subjected to immunobloting analysis of FOXM1, γH2AX, cleaved caspase-3 and β-actin. (B) A2780 and SKOV3 cells with or without silencing of FOXM1 expression, were treated with 1 µg/ml and 2 µg/ml cisplatin for 1 h, respectively. γH2AX foci of A2780 and SKOV3 cells were quantified at different time point: 24, 48 and 72 h after cisplatin treatment. Percentage of γH2AX positive cells was plotted. Untreated cells were used as control.

Given that FOXM1 was overexpressed in ovarian cancer, and was further up-regulated in response to cisplatin treatment, we hypothesized that targeting FOXM1 could sensitize ovarian cancer cell to cisplatin. We transiently transfected two siRNAs targeting FOXM1 in A2780 and SKOV3, both siRNAs remarkably reduced FOXM1 expression and siRNA-2 has a greater silencing effect in the two cell lines (Fig.3A and 3B). 48 h after siRNA-2 transfection, cells were treated with the indicated concentration of cisplatin. Clonogenic assay was performed at 1 h post-cisplatin treatment, and cellular viability was measured using a CCK8 assay at 48 h post-cisplatin treatment. As expected, FOXM1 siRNA transfection in A2780 and SKOV3 cells rendered both cell lines more sensitive to cisplatin toxicity, as evidenced by a comparison of IC_50_ values between scramble siRNA and FOXM1 siRNA-treated cells (∼1.7 µg/ml vs ∼4.1 µg/ml in A2780, ∼2.5 µg/ml vs ∼6.1 µg/ml in SKOV3) (Fig.3C). Treatment with FOXM1 siRNA and cisplatin also resulted in significant reduction in A2780 and SKOV3 cell numbers as measured by clonogenic assay (∼17% vs ∼45% in A2780, ∼16% vs ∼37% in SKOV3) (Fig.3D). Additionally, we examined whether knockdown of FOXM1 led to increased apoptosis after cisplatin treatment. As shown in Fig.3E, cisplatin combined with FOXM1 siRNA resulted in increased apoptosis rates in both cell lines (∼18% vs ∼38% in A2780, ∼20% vs ∼40% in SKOV3). Corresponding with apoptosis assay, western blot analysis also showed enhanced cleaved caspase-3 after co-treatment with FOXM1 siRNA and cisplatin (Fig.4A). Collectively, these results indicate that targeting FOXM1 provides a strategy for sensitizing ovarian cancer to cisplatin.

### 4. FOXM1 knocking-down results in DNA repair deficiency

It has been reported that FOXM1 regulated several genes in the DNA repair pathway[14], [24], we next examined whether FOXM1 knock-down cells were susceptible to DNA breaks in ovarian cancer. To this end, FOXM1 siRNA-treated cells and scramble-treated cells were treated with cisplatin, and western blotting analysis was performed 24 h post-treatment. Notably increased γH2AX was detected in FOXM1 siRNA-treated cells after same treatment with cisplatin (Fig.4A). Furthermore, we stained for γH2AX 24, 48 and 72 h post cisplatin treatment. γH2AX foci per nucleus was counted in more than 100 cells in each time point. The results were expressed as percent of γH2AX positive cells in each time point. FOXM1 siRNA-treated cells displayed high percentage of unprocessed DNA damages (γH2AX positive cells) at 48 h and 72 h after cisplatin treatment when compared to scramble-treated cells (Fig.4B). Therefore, these data support the conclusion of DNA repair deficiency in the FOXM1 siRNA-treated cells.

### 5. Screening for FOXM1 target gene involved in cisplatin-induced DSB repair in ovarian cancer

**Figure 5:**
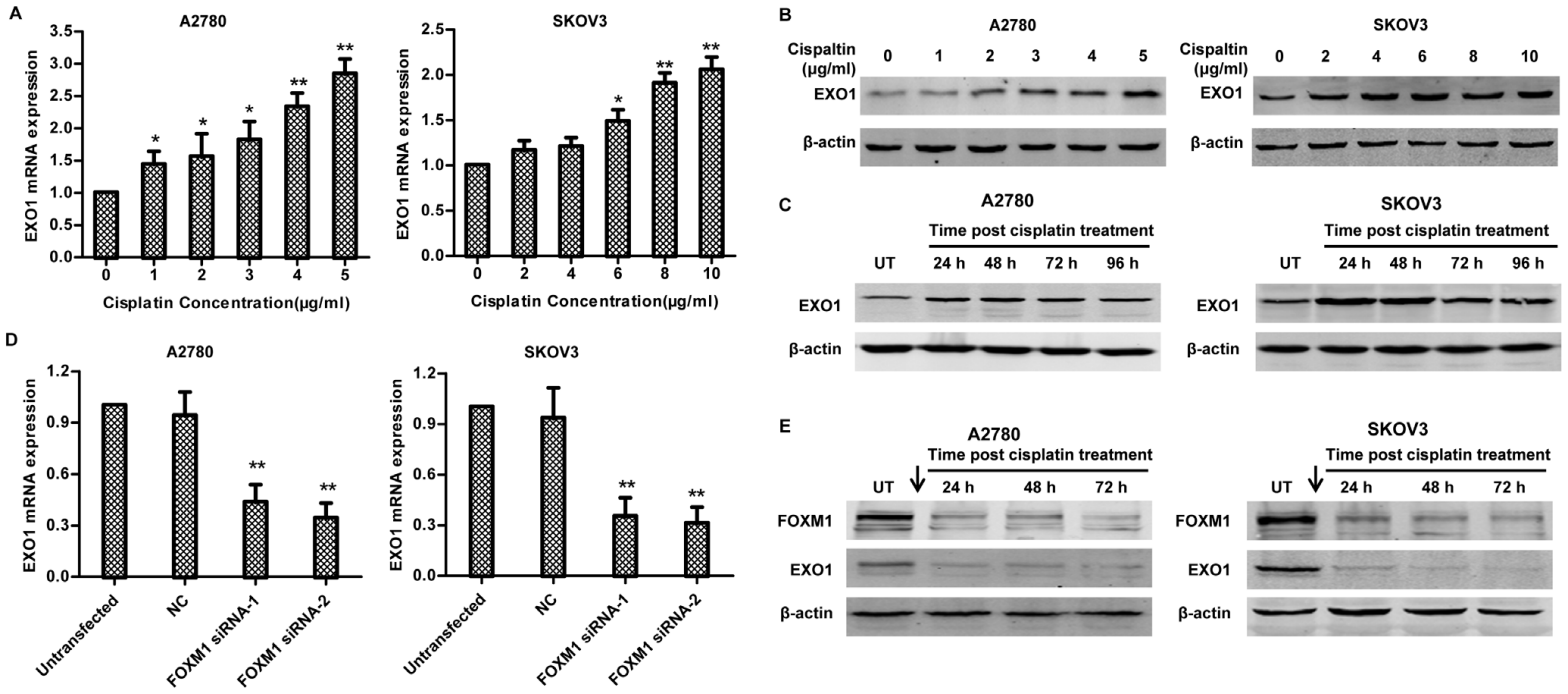
Expression of FOXM1 target genes in response to cisplatin in A2780 and SKVO3 cells. A2780 and SKOV3 cells were treated with the indicated concentration of cisplatin for 24-time PCR analysis (A) and western blotting (B). Results are shown from three independent experiments in triplicates. Columns, mean; bars, SD. (C) A2780 and SKOV3 cells were treated with 2 µg/ml and 4 µg/ml cisplatin for 12 h respectively, then were cultured in fresh complete medium for indicated time points (the time when complete medium was added was set as 0 h). Western blot was performed to determine the expression of EXO1 and β-actin. (D) A2780 and SKO3 cells were transfected with FOXM1 siRNA, 48 h later, EXO1 expression was determined by real-time PCR analysis. Results are shown from three independent experiments in triplicates. Columns, mean; bars, SD. (E) 24 h after FOXM1 siRNA transfection, A2780 and SKOV3 cells were treated with 2 µg/ml and 4 µg/ml cisplatin for 12 h respectively, then were cultured in fresh complete medium for indicated time points (the time when complete medium was added was set as 0 h). The arrow (↓) indicates transfection and subsequent cisplatin treatment. Western blot was performed to determine the expression of FOXM1, EXO1 and β-actin.

Several target genes of FOXM1, such as *BRCA2*, *XRCC1*, *Rad51*, *EXO1*, *PLK4*, has been reported to be involved in DNA repair after the DNA double strand breaks (DSBs)[14], [24]. These results prompted us to determine whether these FOXM1 target genes mediate FOXM1-dependent DNA repair in ovarian cancer. QPCR was employed to determine the expression profile of the above genes after cisplatin treatment. *EXO1* mRNA showed the most robust change and corresponded with the change of FOXM1 protein after cisplatin treatment in A2780 and SKOV3 (Fig.5A). The other genes, however, were slightly or moderately induced after the same treatment (data not shown). Interestingly, we also found that EXO1 protein was not only highly expressed in A2780 and SKOV3 compared to ES2 cell (Fig.S2A), but was up-regulated in a dose and time dependent fashion in A2780 and SKOV3 when treated with cisplatin, just like FOXM1 protein (Fig.5B and Fig.S2B). ON/OFF treatment of cisplatin also resulted in increased expression of EXO1 till 96 h (Fig.5C). It was not surprising that knockdown of FOXM1 was accompanied by 60–70% reduction in EXO1 mRNA expression (Fig.5D). However, FOXM1 knocking-down did not result in profound cell cycle arrest (Fig.S2D), suggesting that reduction of EXO1 was not an indirect effect of cell cycle change. In addition, FOXM1 silencing not only attenuated EXO1 protein expression in response to cisplatin treatment (Fig.S2C), but also after ON/OFF cisplatin treatement (Fig.5E). These results indicate that EXO1 is a candidate target gene of FOXM1 in DNA repair pathway in ovarian cancer.

### 6. FOXM1 directly binds to the EXO1 promoter and regulates its activity

**Figure 6:**
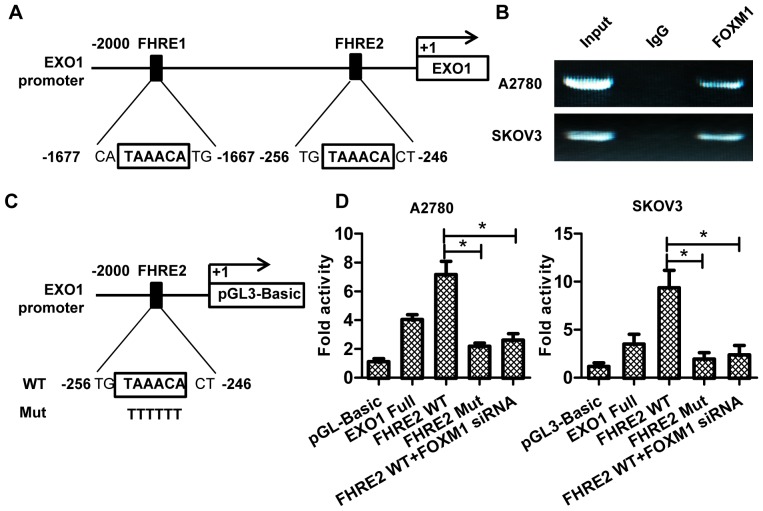
FOXM1 induces the transcriptional activity of the human EXO1 gene through a consensus FHRE site. (A) Sequence and position of the consensus FHRE sequence on the EXO1 promoter. (B) ChIP assays were done in A2780 and SKOV3 cells. Chromatin fragments of the cells were immunoprecipitated with anti-FOXM1 antibody and negative control IgG and subjected to PCR. 1% of the total cell lysates were subjected to PCR before immunoprecipitation as inputs. (C) Schematic structure of wild-type (WT) and mutant (Mut) forms of FHRE2-containing promoter reporters. (D) Luciferase activity with or without mutation in EXO1 promoter. A2780 and SKOV3 cells were transfected with wild-type or mutant FRHE2-containing reporter, or co-transfected with FOXM1 siRNA and wild-type reporter (pGL-3-Basic and pGL3-EXO1 were used as negative and positive control, respectively), then treated with cisplatin for 24 h. Afterwards, luciferase activity in cells were measured. Three independent experiments were conducted.

We postulated that FOXM1 could enhance EXO1 transcription in response to cisplatin treatment. Sequence analysis identified two consensus Forkhead response elements (FHREs) in the promoter region (Fig.6A)[25], [26]. To demonstrate that FOXM1 directly binds to endogenous EXO1 promoter sequence after cisplatin treatment, we performed chromotatin immunoprecipitation assays in A2780 and SKOV3 cells. The anti-FOXM1 antibody, but not the control antibody (IgG), precipitated the FHRE2-containing region (−459/−74) (Fig.6B), however, the FHRE1-containing region (−1934/−1575) was not precipitated (data not shown). To assess the functional role of the FHRE2 in EXO1 regulation, we performed site-specific mutagenesis within FHRE2 of EXO1 promoter pGL3-FHRE2 (Fig.6C). As shown in Fig.6D, mutation of the FHRE2 abrogated the ability of FOXM1 to activate the reporter construct after cisplatin treatment in A2780 and SKVO3, in addition, co-transfection of FOXM1 siRNA and wild-type pGL3-FHRE2 also resulted in low luciferase activity. Interestingly, pGL3-FHRE2 has stronger transcriptional activity than full promoter sequence. These results suggest that FHRE2 mediates the transcriptional effect of FOXM1 on the EXO1 promoter and that there might be some other sites which can be recognized by transcription inhibiting factors in the upstream region of FHRE2.

### 7. DNA repair regulation of FOXM1 is partially mediated by EXO1

**Figure 7:**
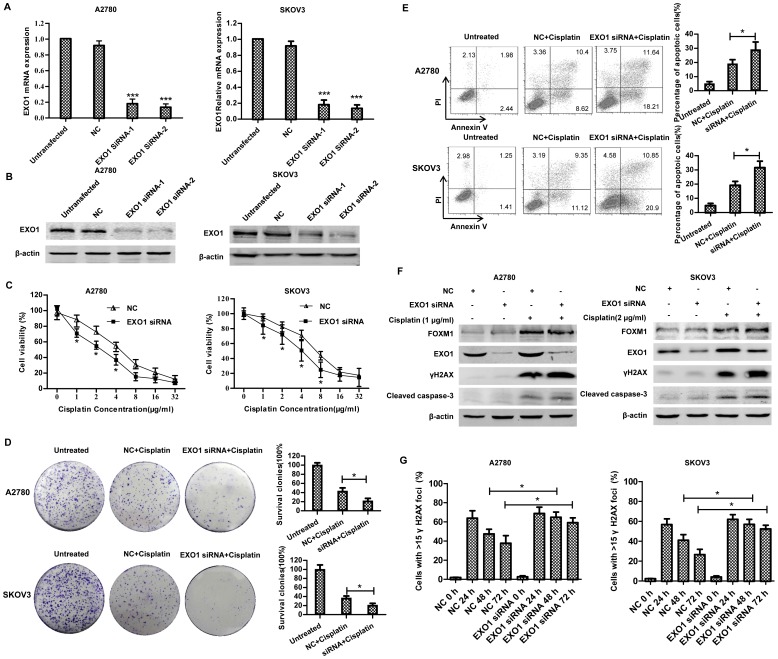
DNA repair regulation of FOXM1 is partially mediated by EXO1. A2780 and SKOV3 cells were transfected with EXO1 specific siRNA or NC siRNA. (A)The expression of EXO1 was determined by real-time PCR analysis 48 h after transfection. (B) Western blotting analysis was done to determine the expression level of EXO1. (C) 48 h after transfection, A2780 and SKOV3 cells were treated with increasing concentrations of cisplatin for another 48 h and their rates of viability were measured by CCK8 and compared to cells without cisplatin treatment. (D) 48 h after siRNA transfection, A2780 and SKVO3 cells were treated for 1 h with 1 µg/ml and 2 µg/ml cisplatin, respectively. Then cells were plated in 6-well plates and incubated for 8–10 d. Then colonies were stained and counted. Results shown are representative of three independent experiments. Graphs provide quantification as a percentage of the nontreated wells. Each column and bar represents mean±s.d. of triplicate determinations. (E) 48 h after transfection, A2780 and SKOV3 cells were treated for 48 h with 1 µg/ml and 2 µg/ml cisplatin, respectively. After treatment, apoptosis was determined by flow cytometric analysis of Annexin V and PI staining. The right panel shows means±s.d. of three independent experiments. (F) 48 h after transfection, A2780 and SKOV3 cells were treated for 24 h with 2 µg/ml and 4 µg/ml cisplatin, respectively. After treatment, cell lysates were prepared, resolved by SDS-PAGE and subjected to immunoblotting analysis of FOXM1, EXO1, γH2AX, cleaved caspase-3 and β-actin. (G) A2780 and SKOV3 cells with or without silencing of EXO1 expression, were treated with 1 µg/ml and 2 µg/ml cisplatin for 1 h, respectively. γH2AX foci of A2780 and SKOV3 cells were quantified at different time point: 24, 48 and 72 h after cisplatin treatment. The percentage of γH2AX positive cells were plotted. Untreated cells were used as control.

EXO1 has been proven to be directly involved in DNA repair mechanism[18], [27], [28]. Thus, we next explored whether EXO1 accounts for FOXM1-mediated cisplatin resistance. We transiently knocked down the expression of EXO1 in A2780 and SKOV3 cells by siRNA transfection. Both siRNAs targeting EXO1 effectively reduced mRNA and protein expression in A2780 and SKOV3 cell lines (Fig.7A and 7B). Knockdown of EXO1 also sensitized cells to cisplatin, as evidenced by a comparison of IC_50_ values (∼2.3 µg/ml vs ∼4.2 µg/ml in A2780, ∼4.1 µg/ml vs ∼6.4 µg/ml in SKVO3) and clone numbers (∼23% vs ∼42% in A2780, ∼21% vs ∼38% in SKOV3) between scramble siRNA and EXO1 specific siRNA-treated cells (Fig.7C and 7D). In accordance with the above, the same treatment with cisplatin resulted in more apoptotic cells in EXO1 specific siRNA-transfected cells (∼18% vs ∼27% in A2780, ∼21% vs ∼33% in SKVO3) compared to scramble-treated cells (Fig.7E). Although EXO1 knock-down did not affect the expression of FOXM1, it did partly recapitulate the cisplatin sensitization effect of FOXM1 silencing in ovarian cancer cells. EXO1 siRNA-treated cells also showed more DNA damages, enhanced apoptosis signaling pathways and impaired DNA repair efficacy, as detected by immunoblotting for γH2AX and cleaved caspase-3 (Fig.7F), and γH2AX quantification (Fig.7G). These results indicate that DNA repair regulation of FOXM1 is at least partially mediated by EXO1.

## Discussion

FOXM1 transcription factor is a regulator of a variety of biological processes including cell cycle progression, apoptosis, angiogenesis, tissue homeostasis and DNA repair[24], [29]. Elevated FOXM1 expression has been reported in many tumor types including ovarian cancer[5], [11]. These findings suggest that FOXM1 plays a key role in tumorigenesis and is a good therapeutic target for human cancer[30]. We explored whether FOXM1 plays any role in modulating cisplatin sensitivity in ovarian cancer in vitro. We show that FOXM1 is up-regulated in chemoresistant ovarian cancer compared to chemosensitive ovarian cancer (Fig.1). Besides, treatment with cisplatin stimulates FOXM1 expression (Fig.2), and gene silencing of FOXM1 sensitizes ovarian cancer cells to cisplatin (Fig.3), through blocking the activation of the DNA repair pathway (Fig.4). We discover that EXO1 is a potential downstream gene of FOXM1 in ovarian cancer (Fig.5), FOXM1 directly binds to EXO1 promoter and enhances EXO1 expression (Fig.6). Finally, we demonstrate that EXO1 plays an important role in the DNA repair pathway activated by FOXM1 in ovarian cancer (Fig.7). Our studies therefore uncover that the FOXM1/EXO1 axis protects ovarian cancer cells after cisplatin treatment by enhancing the DNA damage repair pathway.

In response to DNA damage, the checkpoint network which contains ATM, ATR, chk1 and chk2, is activated and subsequently phosphorylated the downstream proteins, resulting in cell cycle arrest, DNA damage repair, and apoptosis induction[31], [32]. In response to DNA damage caused by IR or UV irradiation, FOXM1 can be directly phosphorylated by chk2, and this modification leads to FOXM1 protein stabilization[23]. In this study, we discovered that FOXM1 expression was elevated at both the transcriptional level and the protein level, and that the elevation of FOXM1 protein was more significant than that of FOXM1 mRNA. This result might indicate that phosphorylation and stabilization of FOXM1 by chk2 contributes largely to FOXM1 expression elevation after cisplatin-induced DNA damage.

Cisplatin is a commonly used chemotherapeutic agent against ovarian cancer. Cisplatin treatment caused intrastrand and interstrand DNA crosslinks (ICLs) formation and DSBs[33], triggering a subset of DNA repair machinery, such as nucleotide excision repair and homologous recombination pathways[34], [35]. Recent study also showed that EXO1 is involved in the repair of DSBs [18]. Our data suggests that EXO1 mediate FOXM1-activated DSB repair in ovarian cancer. FOXM1 regulates XRCC1 and BRCA2 in HER positive breast cancer [14], in triple-negative breast cancer, however, FOXM1 transactivates EXO1 and PLK4 in response to doxorubicin[24]. Besides, targeting FOXM1 also sensitized resistant glioblastoma cells to temozolomide by downregulating Rad51[36]. These studies show FOXM1 can regulate multi-steps of the DNA repair pathway in a context dependent manner in different cancer cells.

Intense research conducted during the past years has revealed that multiple mechanisms account for the cisplatin resistant phenotype of tumor cells, including increased efflux of cisplatin, enhanced ability to repair adducts, and evasion of apoptotic pathways in resistant cells [37]. FOXM1 regulates a variety of downstream genes involved in DNA repair and anti-apoptotic pathway[10]; therefore, it would be more effective to target oncogenic transfactor FOXM1 than targeting only the DNA repair pathway. In accordance with this hypothesis, we found that FOXM1 knockdown seems more efficient than EXO1 silencing in sensitizing ovarian cancer cells to cisplatin treatment.

Cisplatin has been proven to have many adverse effects such as nephrotoxicity, neurotoxicity, ototoxicity [38]–[40]. Novel treatments, such as FOXM1 inhibitor co-treatment with platinum, are potential therapeutic strategies to reduce the necessary dosage of cisplatin and enhance the therapeutic efficacy in treating ovarian cancer. Since FOXM1 is a key regulator of cisplatin response in ovarian cancer, FOXM1 could be a new therapeutic target in ovarian cancer, and inhibition of FOXM1 would overcome cisplatin resistance. In the future, we would like to investigate whether cisplatin combined with FOXM1 inhibitor (thiostrepton or siomycin A) may be good choice to enhance therapeutic response. However, it has been reported that effective inhibition of FOXM1 by thiostrepton requires p53(wild-type or mutated)[12]. These results may indicate that an inhibitor of FOXM1 should be used individually according to p53 status in ovarian cancer.

In conclusion, we found that FOXM1 directly regulated EXO1 expression to promote the DNA repair pathway upon cisplatin treatment, and demonstrated that FOXM1 knockdown can enhance sensitivity of ovarian cancer cells to cisplatin. Thus, FOXM1 might be explored as a candidate of therapeutic target for modulating cisplatin sensitivity in ovarian cancer.

## Supporting Information

Figure S1 **FOXM1 expression at different time point after cisplatin treatment.** A2780 and SKOV3 cells were treated with 1 µg/ml and 2 µg/ml cisplatin respectively, cell lysates were collected at the indicated time point and western blot analysis was performed to determine the protein expression levels of FOXM1, γH2AX and β-actin.
(TIF)

Figure S2 **EXO1 expression after cisplatin treatment and FOXM1 knocking-down.** (A) EXO1 protein in different cell lines was analyzed by western blotting, β-actin was used us endogenous control. (B) A2780 and SKOV3 were treated for the indicated time with 1 µg/ml and 2 µg/ml cisplatin, respectively. EXO1 were examined by western blot after treatment. (C) A2780 and SKOV3 cells with or without FOXM1 silencing were treated with the indicated concentration of cisplatin for 24 h. After treatment, cell lysates were prepared, resolved by SDS-PAGE and subjected to western blot analysis of EXO1 and β-actin. (D) A2780 and SKO3 cells were transfected with FOXM1 siRNA or negative control siRNA, 48 h later, cell cycle were analyzed by flow cytometry.
(TIF)
