# Supplementary figures and images for: FOXM1 Modulates Cisplatin Sensitivity by Regulating EXO1 in Ovarian Cancer
Source: PLoS One. 2014 May 13;9(5):e96989. doi: 10.1371/journal.pone.0096989 (PMC4019642; doi:10.1371/journal.pone.0096989)

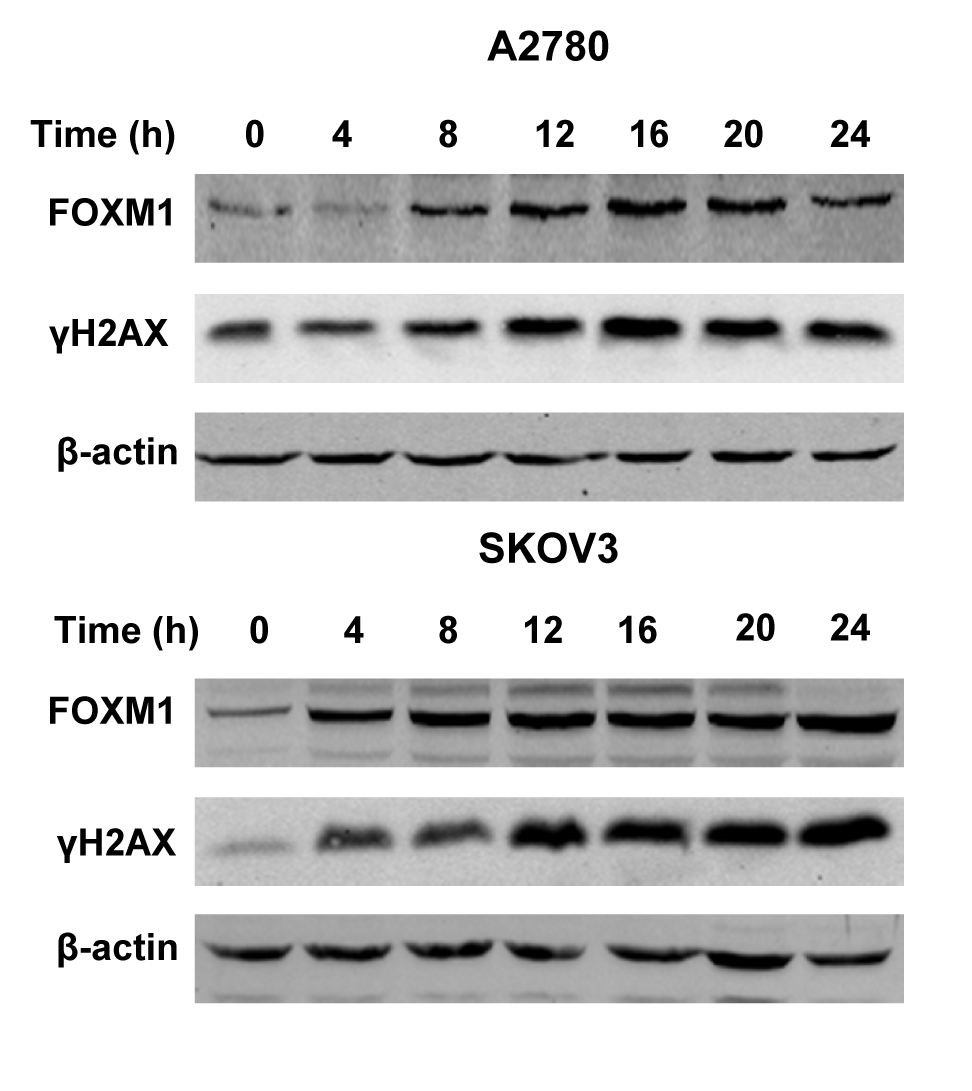

Supplement: Figure S1 — FOXM1 expression at different time point after cisplatin treatment. A2780 and SKOV3 cells were treated with 1 µg/ml and 2 µg/ml cisplatin respectively, cell lysates were collected at the indicated time point and western blot analysis was performed to determine the protein expression levels of FOXM1, γH2AX and β-actin. (TIF) [file pone.0096989.s001.tif]

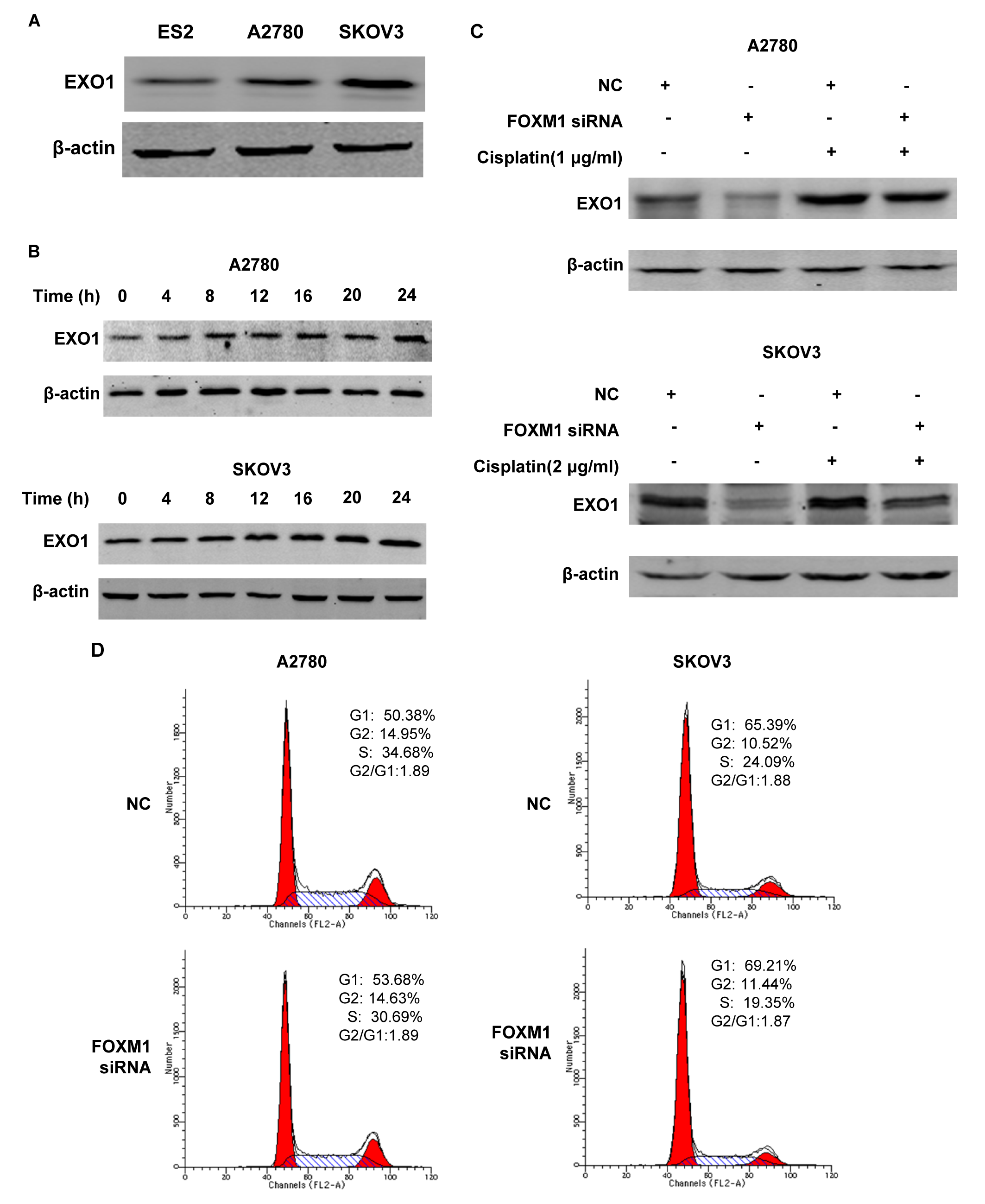

Supplement: Figure S2 — EXO1 expression after cisplatin treatment and FOXM1 knocking-down. (A) EXO1 protein in different cell lines was analyzed by western blotting, β-actin was used us endogenous control. (B) A2780 and SKOV3 were treated for the indicated time with 1 µg/ml and 2 µg/ml cisplatin, respectively. EXO1 were examined by western blot after treatment. (C) A2780 and SKOV3 cells with or without FOXM1 silencing were treated with the indicated concentration of cisplatin for 24 h. After treatment, cell lysates were prepared, resolved by SDS-PAGE and subjected to western blot analysis of EXO1 and β-actin. (D) A2780 and SKO3 cells were transfected with FOXM1 siRNA or negative control siRNA, 48 h later, cell cycle were analyzed by flow cytometry. (TIF) [file pone.0096989.s002.tif]
